# Life Cycle Environmental Impacts of Sewage Sludge Pyrolysis and Their Dynamic Evolution

**DOI:** 10.1021/acsenvironau.5c00097

**Published:** 2025-10-27

**Authors:** Jan Matuštík, Aleš Paulu, Jaroslav Moško, Michael Pohořelý

**Affiliations:** † Department of Sustainability and Product Ecology, University of Chemistry and Technology, Prague Technická 5, Prague 160 00, Czech Republic; ‡ Charles University Environment Centre, José Martího 407/2, Praha 6 160 00, Czech Republic; § Department of Power Engineering, Faculty of Environmental Technology, University of Chemistry and Technology, Prague Technická 5, Prague 6 166 28, Czech Republic

**Keywords:** sewage sludge treatment, biochar, life cycle assessment, prospective LCA, dynamic LCA

## Abstract

Thermal methods, especially pyrolysis with biochar production, are emerging as potential solutions for sewage sludge treatment. Life cycle assessment (LCA) is commonly used to evaluate environmental impacts, and the promising performance of pyrolysis has been demonstrated in previous LCA studies. This study goes into further detail in impact analysis by applying prospective and dynamic LCA while incorporating multiple approaches to consider biogenic carbon emissions. The results show that the system provides climate benefits over a 100 year period, with findings remaining robust despite variability in facility parameters and uncertainties in model assumptions. The prospective LCA results indicate that the climate balance of the system is expected to improve over the years. The dynamic analysis demonstrates that the system provides significant temporal carbon capture, which gradually decreases as biochar decomposes in soil. Taking two perspectives on biogenic carbon accounting reveals how the results can be affected by methodological decisions. This study offers a more detailed view of the dynamic evolution of climate impacts across the facility’s entire operational lifetime.

## Graphical abstract

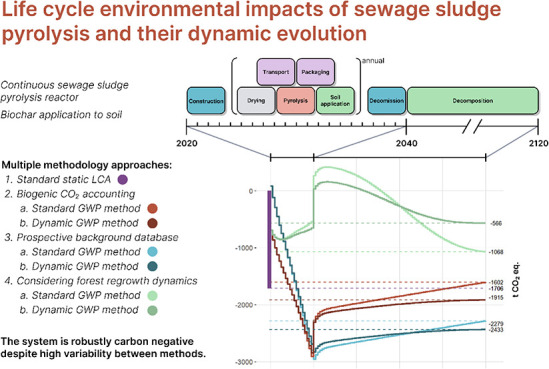

## 1 Introduction

With the climate crisis looming large,^1^ the need for greenhouse gas (GHG) emission reduction and carbon capture is ever more pressing. In this situation, it is imperative to address all sources of emissions, including those from wastewater treatment. Wastewater treatment generates significant volumes of sewage sludge, which requires proper management to curb environmental and health risks.^2^ Traditional sludge treatment methods such as direct agricultural use, composting, or landfilling are becoming less viable due to concerns over contamination, nutrient loss, or misalignment with circular economy principles.^3^ In response, thermal treatment methods are emerging as efficient alternatives, though further research is required about their environmental impacts.^4,5^

The standard approach to assess and compare environmental impacts is the Life Cycle Assessment (LCA) methodology.^6^ The methodology has already been applied to assess and compare the available options for sewage sludge treatment. Chang et al.^7^ applied LCA to compare conventional sludge treatment technologies, considering the effect of variability and parameter distribution on climate impacts. They identified significant uncertainty in the impact results with thermal drying and incineration being the only technological option standing out as significantly better. However, sludge pyrolysis is not among the assessed options in that study.^7^ Pyrolysis of sludge and biochar production has been an emerging option in the past years; hence, the details of the available studies vary. Many studies analyze only climate impacts in a simplified manner,^8,9^ and most studies are based on laboratory, modeling, or literature estimates of the properties of sludge pyrolysis.^9–12^ However, the robustness and detail of available LCA studies have improved in the past years. Chang et al.^13^ compared the climate impacts of emerging sludge treatment technologies and evaluated the uncertainty in the results driven by variance in model parameters. Recently, there have also been published several studies based on real-life data of sludge treatment.^14,15^ Most studies indicate that thermal methods, especially methods providing biochar, have high potential for climate impact savings.

Nevertheless, the climate balance of sludge biochar production depends on many factors like feedstock, process parameters,^13^ as well as the assumption about biochar decomposition.^16^ With sewage sludge pyrolysis, a large fraction of impacts is usually driven by the process of sludge drying.^11^ Therefore, the environmental impact depends on the background source of energy and its evolution in time. Furthermore, the results of biochar LCAs also depend on many methodological decisions and assumptions, especially regarding the treatment of biogenic carbon.^17^ The claim of carbon capture with biochar hinges mainly on the assumption of avoided energy production and the behavior of biochar in soil. Nevertheless, biochar decomposition in soil is a dynamic process, and a fraction of the storage is only temporary. The standard LCA practice is to sum all of the emission pulses into one, disregarding when it happens. However, at the time when there is an imminent risk of crossing climate tipping points,^18^ the timing of emission could be of significance. Those elements and considerations are currently missing in the LCA literature on sewage sludge pyrolysis systems, which limits the robustness of the findings.

This study aims to fill this gap by evaluating from multiple methodological perspectives, the life cycle environmental impacts of sludge pyrolysis with the application of biochar to soil. Next to the standard LCA method, prospective LCA modeling is used to investigate how future changes in the background system are expected to affect the climate balance. The dynamic emission profile of the system is assessed by using dynamic inventory to show the temporal evolution of the impacts. Dynamic characterization is employed to evaluate the climate impacts over a 100 year horizon. Furthermore, the effects of various perspectives on biogenic carbon accounting are evaluated. The results derived using standard LCA methodology are compared with those of alternative methodologies, and the effect of those methodological decisions is discussed.

## 2 Methods

### 2.1 System Description

The system boundaries of the study are listed in Figure 1. The modeled system starts with dewatered sewage sludge entering the dryer, while the prior processes of wastewater treatment are outside of the system boundaries. The dewatered sludge (24% dry matter content) is dried using the heat recuperated from the pyrolysis process (supplying over 40%) in combination with heat from a central heating system. The dried sludge (90% dry matter content) then enters the pyrolysis unit. The pyrolysis unit consists of a continuous screw-type reactor. The primary pyrolysis gas (a mixture of condensable vapors and permanent gases) is incinerated in the combustion chamber without prior condensation. The heat from combustion is utilized via a tube-in-tube system to indirectly heat the pyrolysis process, achieving a pyrolysis temperature of 650 °C inside the reactor. A detailed description of the facility and biochar properties is provided in ref. 19. The produced biochar is packaged into standard 1 tonne polypropylene bags and transported to be applied into agricultural soil. The default transportation scenario corresponds to the common situation in the Czech Republic. The system boundaries contain the construction of the facility as well as its decommission. The lifetime of the operation is expected to be 20 years.

**Figure 1:**
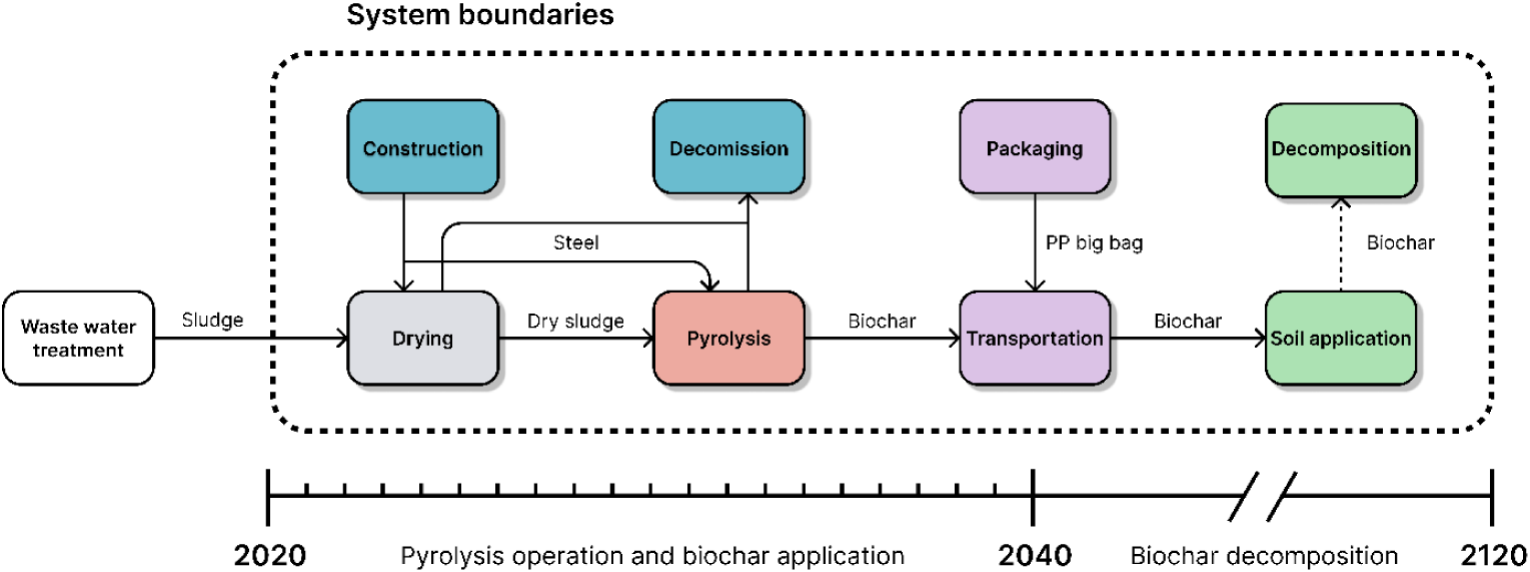
Visualization of the system boundaries and the temporal evolution of the system.

While the drying, pyrolysis, and biochar properties are based on measurements in a real sludge treatment facility in Trutnov, Czech Republic, the model of biochar application to soil is based on the general literature and model estimates. The behavior of biochar in soil is highly variable and depends on the biochar properties, soil, and climate conditions. As the goal is to evaluate a general status rather than a particular situation, for the purpose of the study, it is more robust to apply generalized values rather than specific experimental measurements. The stability of biochar in soil over 100 years is estimated at 71% using the IBI biochar calculator.^20^ Considering the uncertainty in this estimate,^21^ a normal distribution with a standard deviation of 10% is assumed.

One of the benefits of sludge pyrolysis is the potential for recycling some of the nutrients (N, P, and K) contained in wastewater. When the focus of an LCA study is on the effect on agricultural production, nutrient recycling is usually captured by assuming a set reduction in fertilizer application rate.^22,23^ In contrast, agricultural processes are not the focus in this study. Hence, the quantification approach is simplified based on the assumption that the nutrients contained in biochar directly substitute mineral fertilizer based on the content of individual nutrients in the biochar. However, a fraction of the nutrients is stably incorporated in the biochar structure and thus is not utilized by plants in the time horizon of the study. The baseline assumption is that 50% of the nutrients contained in biochar are mobilized and substitute mineral fertilizers. A wide uncertainty range of 20–80% (triangular distribution) is applied to account for the high variability and uncertainty in this value. The rest of the material is assumed to remain stable and inaccessible to plants.

The effect of biochar application on N_2_O emission rate is highly variable, depending on biochar properties, soil type, crop, or fertilization rate.^24–27^ Based on a meta-analysis of experimental results,^25^ the baseline assumption here is that biochar application leads to 30% N_2_O emission reduction, with the uncertainty interval from 40% reduction to 20% increase (triangular). The avoided emissions are calculated based on the FAO (Food and Agriculture Organization) emission factors for temperate croplands^28^ assuming a 20 t ha^–1^ biochar application rate, and the emission reduction effect lasting for a single season after application (eq 1).

1 $$N_{2}O_{avoided}=\frac{N_{2}O\;emission\;rate\cdot Emission\;reduction\;factor}{Biochar\;application\;rate}$$

The experimental results are less decisive for the effect on methane emissions, where meta-analyses suggest both positive and negative effect scenarios with the mean around zero.^27,29^ Therefore, the potential effect of biochar application on soil methane emissions was not included in the analysis.

### 2.2 Standard LCA

Life Cycle Assessment (LCA) was conducted following the principles of international standards.^30^ The study employed a generation-based functional unit representing the entire operational lifetime of the facility. Furthermore, results were quantified in relation to the functional unit of 1 year of operation and with unitary-based functional units of 1 tonne of dewatered sludge and 1 tonne of biochar. As outlined in the previous section, substitution modeling was applied to account for the nutrients in biochar, replacing average market-available mineral fertilizers. The foreground system is based on operational parameters and direct measurements at the sludge treatment facility. Background data were sourced from the ecoinvent 3.9 database (attributional, cutoff).^31^ The parameters of the system, their uncertainty distributions, and the database processes used in the LCA study are available in Supporting Information.

Impact assessment was conducted using the Environmental Footprint (EF) 3.1 methodology^32^ which includes 16 impact categories covering ecosystem quality, human health, and resource consumption. The methodology also provides normalization and weighting factors, enabling the conversion of impact results into unitless, comparable indicators. Particular attention was given to GHG emissions and storage, expressed in CO_2_ equiv. The uncertainty range of the results was evaluated using Monte Carlo simulation with 10,000 simulations drawing random samples from the uncertainty distribution of the parameters.

### 2.3 Prospective LCA

Prospective LCA enables the modeling of the evolution of the background system driven by technological progress or public policy. The prospective changes in the environmental impacts are modeled by coupling traditional LCA background inventory databases with scenarios from the Integrated Assessment Models (IAMs), capturing expected technological and economic shifts. This study utilizes the *Premise* tool developed in ref. 33 to integrate the ecoinvent 3.9 database with the REMIND IAM. The IAM provides the results for multiple Shared Socioeconomic Pathways (SSPs) for global energy and economic systems under varying climate and sustainability constraints.^34^

The SSP2 pathway was selected to represent the “middle of the road” scenario, in which social, economic, and technological trends follow historical patterns without significant deviations. Within this pathway, the No Policy Implemented (NPi) scenario is applied, which assumes no additional climate policies or mitigation measures beyond those currently in place. This scenario corresponds to a radiative forcing of 4.5 W m^–2^, aligning with the Representative Concentration Pathway (RCP) 4.5.^35^ This selection reflects a moderate and pragmatic development trajectory that is realistic, given the current global policy landscape.

The prospective ecoinvent database based on the SSP2-NPi trajectory was generated using the ScenarioLink plugin in the Activity Browser software and builds upon the Brightway2 LCA framework. The resulting database was structured into five-year intervals from 2020 to 2040, aligning with the expected lifetime of the sludge treatment facility.

Heat consumption for sludge drying is a key component of the analyzed technology. However, the ecoinvent database lacks a data set for a European heat consumption mix that includes both fossil and renewable energy sources and is compatible with prospective models in Premise. To address this, a heat consumption mix for the EU-27 region was developed, projecting evolution toward 2050 based on the European Commission’s^36^ baseline forecast for industrial heating and cooling demand. This forecast anticipates moderate decarbonization with a shift primarily toward biomass sources.

### 2.4 Dynamic LCA

In contrast to standard LCA practice, dynamic LCA differentiates the time of the environmental pressure as well.^37^ The dynamic, time-differentiated inventory was developed only regarding GHG emissions, as those are arguably the crucial considerations for the viability of the sludge-biochar systems. Furthermore, the importance of this impact is corroborated by its prominence among the normalized and weighted impact category results (Figure 2). As is standard practice, the time horizon for consideration of climate impacts was 100 years. The temporal evolution of the system is visualized in Figure 1. The temporal system boundaries start with the construction of the pyrolysis unit in 2020 (year 0). The operation starts in year 1 and continues for the next 20 years after which the structure is decommissioned. The biochar is applied to soil as it is produced, until the year 20. After the decommissioning of the technology, the only process that is happening is the decomposition of biochar in the soil. The two-pool model presented in ref. 38 was applied to construct the biochar decay curve (eq 2)

**Figure 2:**
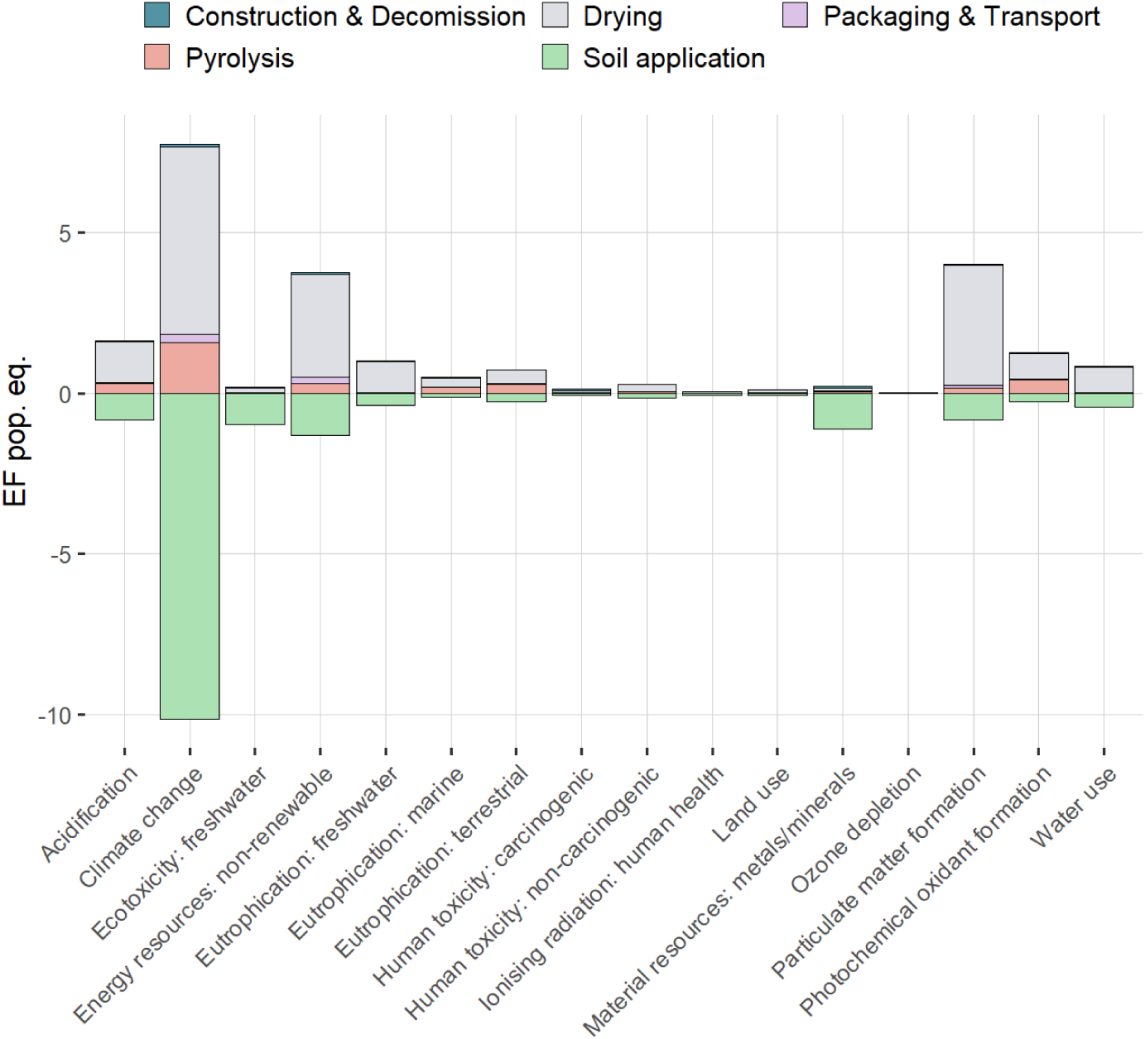
Annual impacts of the analyzed system on the Environmental Footprint 3.1 impact categories, normalized and weighted, analyzed with the standard LCA approach.

2 $$y=a\cdot e^{\left(-k_{1}\cdot t\right)}+b\cdot e^{\left(-k_{2}\cdot t\right)}$$

where *y* represents the remaining fraction of biochar in year *t*, *a* is the size of the labile pool and *b* is the size of the recalcitrant pool, and *k*_1_ and *k*_2_ are the exponential coefficients. The size of the labile pool was set at 20%, and the size of the recalcitrant pool was set at 80% based on the meta-analysis in ref. 39. The values for the exponential coefficients (*k*_1_ = 0.511111, *k*_2_ = 0.001​193​467) were found numerically (using MS Excel Solver) with the condition that the remaining fraction of biochar carbon after 100 years is 71% (i.e., at *t*_i_ = 100 years, *y* = 0.71).

Dynamic characterization of the greenhouse gas emission flows was applied next to the standard GWP100 factors. The dynamic GWP100 (dyn-GWP) characterization factors for the RCP 4.5 middle-of-the-road scenario were calculated in ref 40. The standard practice in LCA is to not consider biogenic carbon,^41,42^ assuming biogenic CO_2_ emissions are a part of the closed system of biomass growth and decomposition. However, plant growth and CO_2_ emission from biomass happen at different time points, and the gap can reach over 100 years in the case of forestry products. While standard LCA methodology does not consider this aspect, it can be crucial with dynamic LCA. Hence, the carbon uptake to biomass and emission from biomass decomposition are tracked in the Biogenic scenario. Furthermore, there are two possible perspectives on biomass uptake. In the basic approach, from the perspective of a product, the carbon uptake to biomass happens in the early stages of the system, before the emission. From another perspective, biogenic CO_2_ emissions result in a temporary increase in atmospheric concentration and radiative forcing, which is balanced only by biomass regrowth. While this difference is marginal with annual and fast-growing plants, it can be crucial for long-lived forestry biomass. Following the GWP-bio methodology,^43,44^ the Forest regrowth scenario considers the biogenic CO_2_ emissions, mainly from heat production for drying, to be compensated for only once the carbon is taken in by trees in a forest. Following,^43^ biomass growth is modeled as a normal distribution (eq 3)­

3 $$g\left(t\right)=\frac{1}{\sqrt{2\pi \sigma^{2}}}\cdot e^{\frac{-{\left(t-\mu \right)}^{2}}{2\sigma^{2}}}$$

where *g* is the fraction of the emission taken up in the year *t*, the mean μ corresponds to half of the rotation period (assumed to be 100 years for spruce forest), and σ is set at μ/2.

## 3 Results

### 3.1 Standard LCA

As shown in Figure 2, the climate change impact category is the most prominent environmental issue affected by the analyzed system. Drying of the dewatered sludge is the main driver of climate impacts, followed by pyrolysis. In comparison, Packaging & Transport and Construction & Decommissioning appear marginal. Biochar application to soil, with carbon capture and avoided emissions, leads to significant climate benefits, which tilt the overall balance into negative numbers. The system is thus climate negative across the entire life cycle. Throughout the entire life of the facility, with 20 years of operation, the system could capture 1.7 Gg of CO_2_ equiv. While the Monte Carlo analysis indicates that there is a substantial uncertainty in the processes of pyrolysis and soil application, more than 90% of the modeled results are in the negative values (Figure 3). As shown in Table 1, the system could capture 85 Mg CO_2_ equiv per year. The life cycle climate benefits of processing 1 tonne of dewatered sludge by pyrolysis are 27.5 kg CO_2_ equiv and the life cycle benefits related to producing 1 tonne of sludge biochar and applying it to soil are 201 kg CO_2_ equiv

**Figure 3:**
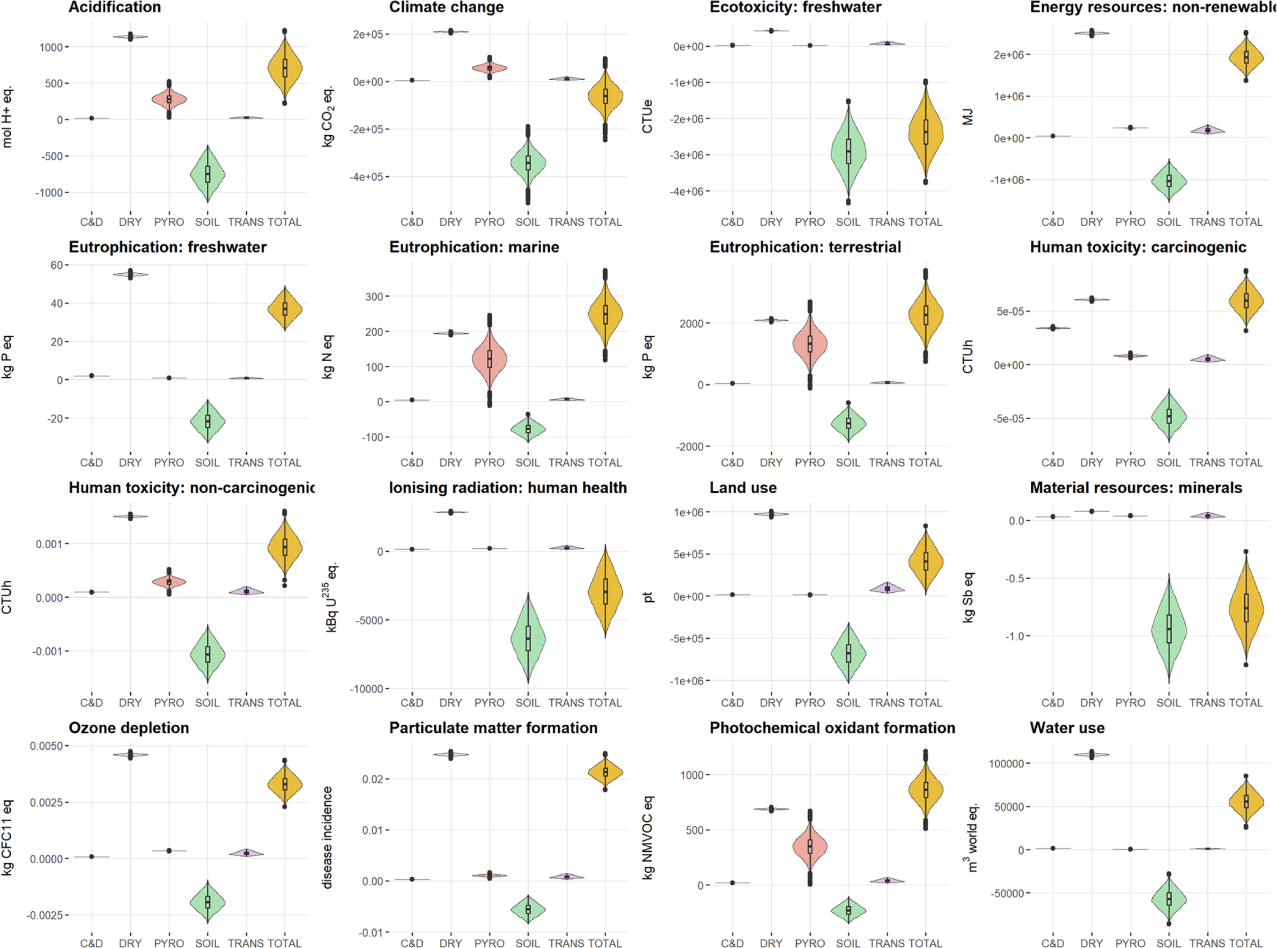
Variability of the EF impact category results, analyzed by the standard LCA and Monte Carlo simulation, per year of operation. C&D = construction and decommissioning; DRY = drying process; PYRO = pyrolysis process; SOIL = application of biochar to soil; TRANS = packaging and transportation of biochar; TOTAL = total impact of the system.

**Table 1:** Comparison of Climate Impacts in kg CO_2_ eq between the Different Methodological Approaches and for Different Functional Units: The Entire Lifespan of the Facility; 1 year of Operation; 1 tonne of Produced Biochar; and 1 tonne of Processed Dewatered Sewage Sludge^a^

	Lifespan	1 year	1 t biochar	1 t sludge
	kg CO_2_ equiv	kg CO_2_ equiv	kg CO_2_ equiv	kg CO_2_ equiv
Standard	–1,706,448	–85,322	–201	–27.5
Prospective	–2,273,557	–113,678	–267	–36.7
Biogenic	–1,602,268	–80,113	–189	–25.8
Forest regrowth	–1,067,719	–53,386	–126	–17.2
Prospective dyn-GWP	–2,432,804	–121,640	–286	–39.2
Biogenic dyn-GWP	–1,914,683	–95,734	–225	–30.9
Forest regrowth dyn-GWP	–566,264	–28,313	–67	–9.1

a Negative values correspond to climate benefits .

Next to Climate change, the most prominent impact categories according to the normalized and weighted results (Figure 2) are the consumption of nonrenewable energy resources and Particulate matter formation, both driven mainly by the life cycle impacts of sludge drying. Sludge drying is the main factor in all of the impact categories. On the other hand, the benefits coupled with the application of biochar to soil are prominent in most impact categories as well. The Monte Carlo analysis (Figure 3) shows a substantial variability of impacts of pyrolysis, caused by the variable conditions in the process and uncertainty in analytical measurements. Similarly, major uncertainty was observed with the benefits of biochar soil application, where there is uncertainty in the amount of avoided fertilizer, the change in N_2_O emissions, and the expected biochar stability over the 100 years. Hence, the total balance of environmental impacts modeled by Monte Carlo is highly variable as well. This uncertainty affects the magnitude of impacts or benefits, but in most cases it does not affect the direction of the results.

Sludge drying was shown to be the major driver of impacts in this system, which calculates with a regional mix of sources of energy. While this average mix is the most representative of general conditions, it masks the variability of the different processes at the level of individual facilities, which usually employ only one of the available drying options. As shown in Figure 4, the impacts of these different energy sources highly differ across impact categories. While using biomass to derive energy for drying has low fossil CO_2_ emissions, and thus a low impact in the standard GWP100 calculation approach, it is coupled with major emissions of biogenic CO_2_, as well as high emissions of particulate matter. On the other hand, burning natural gas has low particulate matter impacts but causes depletion of fossil energy resources. The Other fossils mix dominated mostly by coal or oil products is unequivocally the worst option across most impact categories. The ecoinvent model of future heat production (Biogas) is mostly based on biogas utilization and thus has low impacts. However, it is doubtful that there would be enough biogas available to substitute all of the current energy sources. The real sewage-sludge treatment facility this study is based on currently utilizes energy from biomass for sludge drying. The life-cycle climate benefits of this specific scenario amount to 394 kg CO_2_ equiv per 1 tonne of biochar.

**Figure 4:**
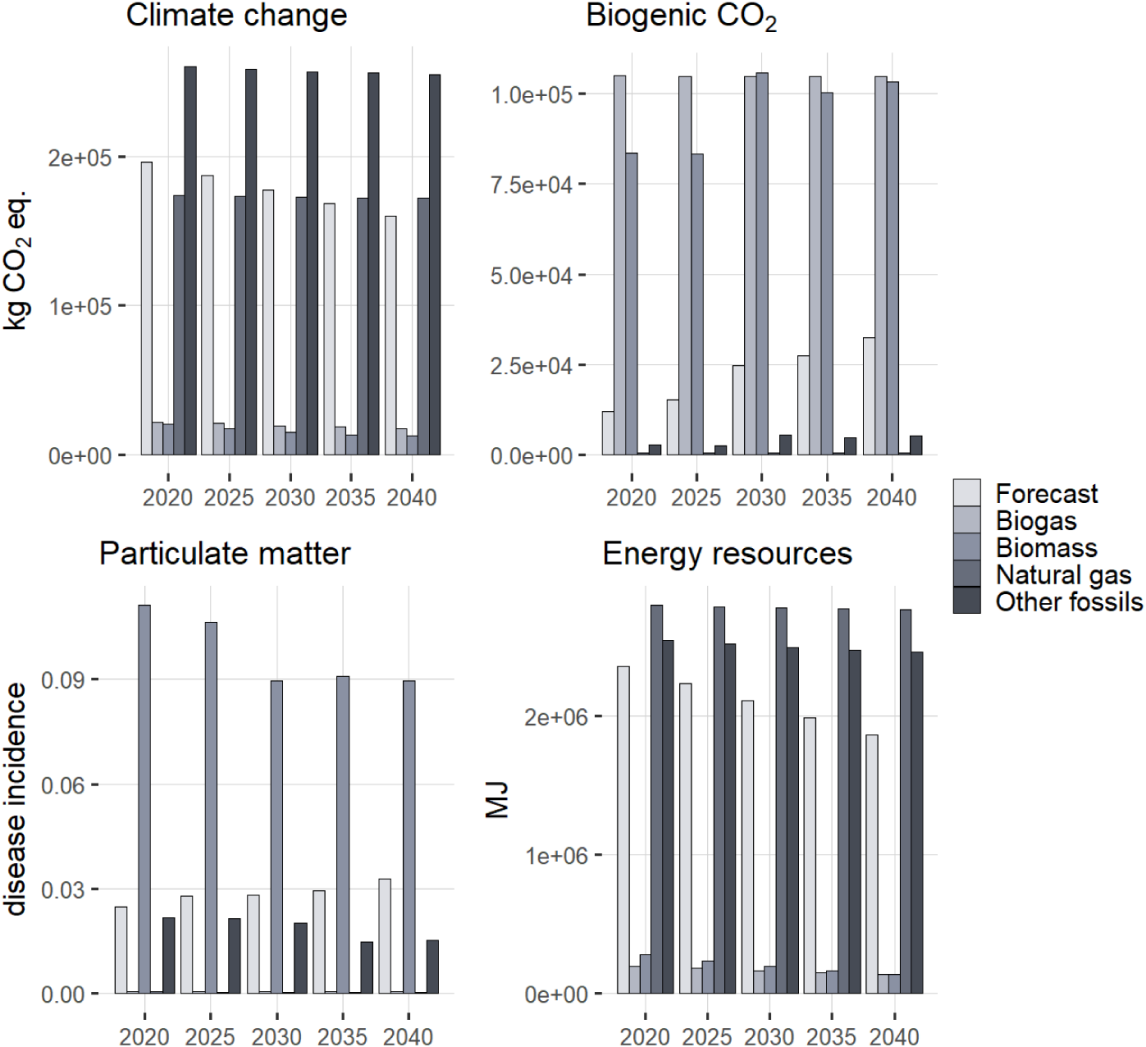
Comparison of annual impacts evaluated using the standard LCA approach for the available heat sources for drying. “Future” corresponds to the ecoinvent future mix, “Biomass” represents mostly wood biomass burning, “Other” represents a mixture of other resources, mostly coal and oil products but also biogas in a small quantity. The “mix” corresponds to the expected mix of energy sources according to EU forecasts.

A general sensitivity analysis was conducted to evaluate how robust the findings are to variation of key parameters (Figure 5). As presented above, the consumption of thermal energy for drying is one of the main sources of impact. Reducing the demand for heat, by increasing efficiency or employing innovative drying methods (see section 4), could bring further benefits. The performance of the system in providing climate benefits could improve by over 2% with 10% efficiency gains and by over 12% when the need for external heating sources is fully eliminated. Changes in biochar transportation distance have only a minor effect on the overall performance. Even with the distance of 500 km, which is rather unlikely in normal circumstances, the climate benefits are reduced by only less than 2%. As indicated in Figure 3, the behavior of biochar in soil is a major source of uncertainty in the results. If the stability of biochar over 100 years is only 50%, the overall climate savings could be reduced by over 4%. On the other hand, if stability is higher, at 80%, the savings are increased by almost 2%. Similarly, if nutrient availability, and thus the mass of substituted mineral fertilizers, is increased to 80%, then the performance of the system could be improved by over 5%. However, if nutrient utilization were only 20%, the climate savings could be reduced by 11%. This sensitivity is driven mainly by the high content of phosphorus in the biochar.

**Figure 5:**
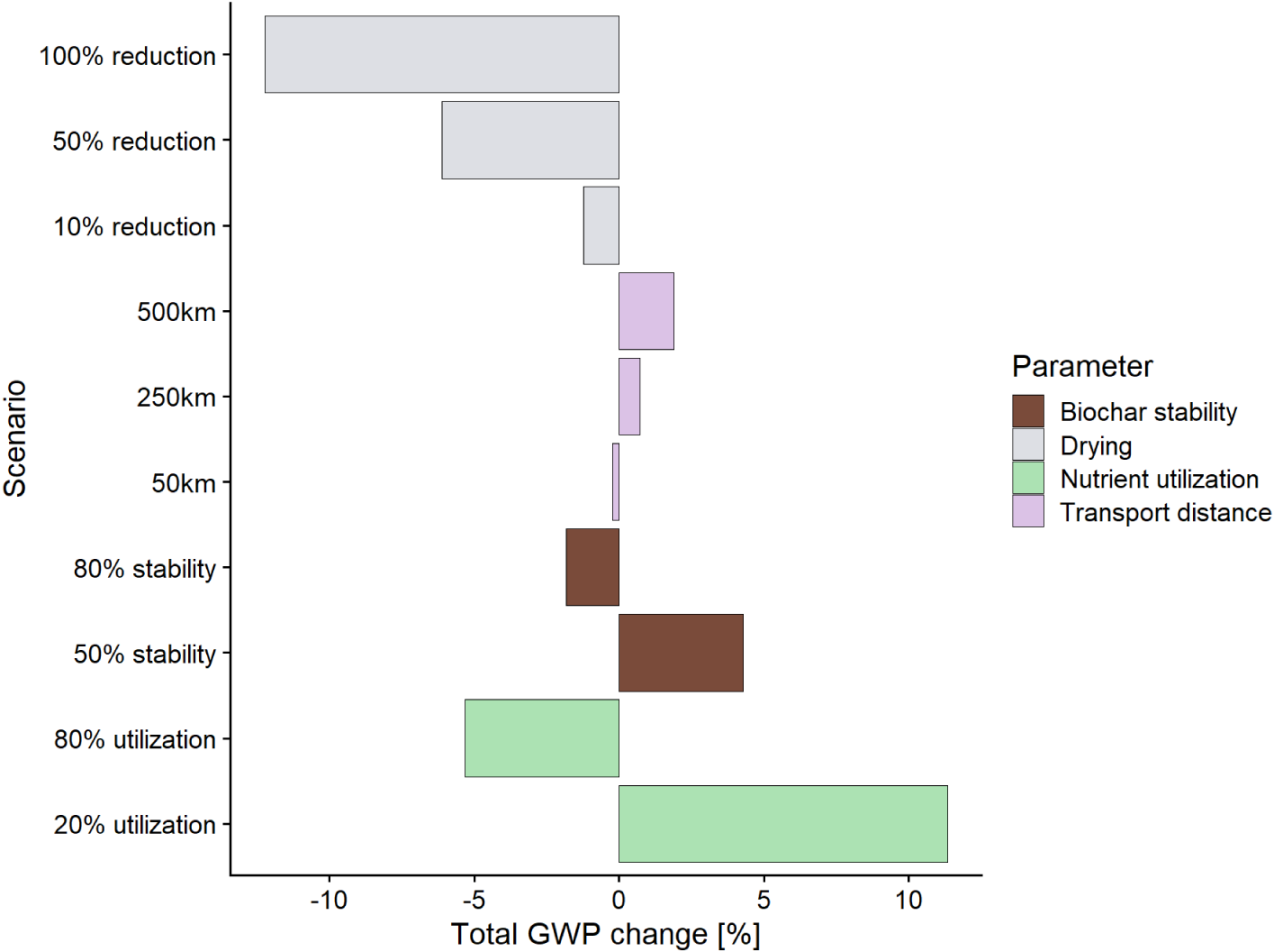
Sensitivity analysis. The effect of variation of key parameters on the overall performance of the system is in the Climate Change impact category.

### 3.2 Prospective and Dynamic LCA

The dynamic prospective results for climate change impacts are visualized in Figure 6. Considering the modeled evolution of the background system, the overall climate benefits of the Prospective scenario are significantly higher than with the standard approach, 2.3 Gg CO_2_ equiv compared to 1.7 Gg CO_2_ equiv. This difference is driven mainly by the evolution of background energy sources for drying (Figure 3), but also by a slight decrease in the impacts of pyrolysis and transportation. On the other hand, the benefits of avoided fertilizer production slightly decrease as their production is expected to become more environmentally friendly. In contrast, considering biogenic emissions results in smaller climate benefits in comparison with the standard approach. Despite the prospective improvements in the system, the overall benefits decrease to 1.6 Gg CO_2_ equiv. As the curve in Figure 6 shows, the first year is marked by a pulse of climate impacts caused by the construction of the facility. In the next 20 years, carbon is accumulated in the system, as carbon captured from biomass and deposited to soil with biochar exceeds the emissions from drying and pyrolysis. In the 20th year, there is no longer carbon capture, but the pyrolysis utilizes the carbon captured in the previous year; hence the pronounced jump. After 20 years, the only impacts of the system are caused by the gradual decomposition of biochar in soil.

**Figure 6:**
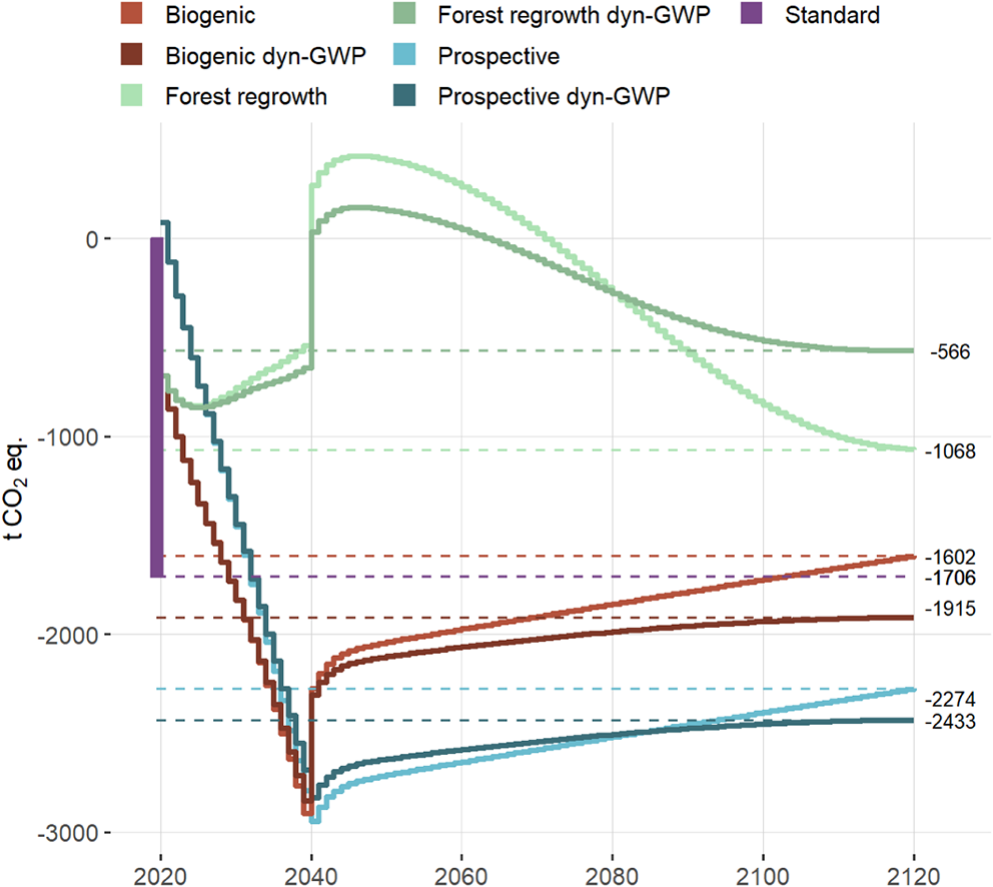
Dynamic evolution of the climate balance of the system over the 100 year time horizon. Comparison of different methodological approaches. Negative values correspond to climate benefits.

The impact curve is different for the Forest regrowth scenario. In the Biogenic scenario, all the carbon in biomass is assumed to be taken up prior to its harvest and oxidation. In contrast, in the Forest regrowth scenario, the carbon in wood biomass used for drying sludge is taken up only later as the forest grows back. Hence, in the first 20 years, the emissions from facility operation are higher than the carbon capture. After 20 years, however, the emissions are gradually compensated for by forest regrowth. Still, this leads to a decrease in the quantified overall climate benefits to 1.1 Gg CO_2_ equiv. Furthermore, in contrast to the Prospective and Biogenic scenarios, the Forest regrowth approach indicates a temporal increase in atmospheric CO_2_ concentration caused by the system. Although the impacts are largely negated over the 100 year period, the temporal increase in radiative forcing could contribute to a crossing of climate tipping points. With static characterization, the effect of utilizing fast-growing woody biomass in contrast to spruce is marginal within the 100 year period. However, increasing the rotation period to 120 years reduces the benefits to 0.8 Gg CO_2_ equiv as a large part of the emissions is not negated over the assessment period.

Applying dynamic characterization adds a further contrast to the results in comparison to the standard approach. Because the dynamic characterization favors later emission times, even the temporal storage of carbon in biochar results in climate benefits. Hence, the overall balance of the Prospective scenario shows higher climate benefits in comparison to static characterization (2.4 Gg CO_2_ equiv), and the same stands for the Biogenic scenario with 1.9 Gg CO_2_ equiv (Figure 6). Dynamic characterization has the opposite effect on the Forest regrowth scenario, where there is a temporal release of carbon rather than temporal storage. The overall benefits are then only about half of those calculated with static characterization, quantified at 0.6 Gg CO_2_ equiv. The effect of a change in rotation period is much more pronounced with dynamic characterization, which differentiates the timing of emission. For wood biomass with a rotation period of 20 years the total benefits of the system were quantified at 1.3 Gg CO_2_ equiv, while for a rotation period of 125 years the benefits are only 0.3 Gg CO_2_ equiv.

## 4 Discussion

The system of sludge pyrolysis with biochar soil application was found to be climate negative, meaning it provides carbon capture and achieves negative CO_2_ emissions^45^ over the 100 year time horizon. This finding appears robust to uncertainty in the system parameters and measurement error captured with Monte Carlo analysis. Nevertheless, the climate balance is sensitive to the heat source used for drying. The system shifts to an overall climate impact forcer in case of use of “dirty” sources like coal or fuel oil represented by the Other category in this study. Furthermore, while natural gas utilization appears to be a significantly more favorable option, it is possible that methane leakage during production is underestimated^46^ and its impacts are in fact higher than indicated here. The use of biomass for drying appears to be the most favorable option, even when considering biogenic carbon, but the competition for biomass energy is expected to increase in the future,^47^ affecting its availability. Hence, searching for alternative, more climate-efficient methods of sludge drying would be the most efficient way to achieve better performance of the system. A promising option is the use of solar dryers, which could enable to dry the sludge with minimal environmental impacts and even free up a part of the heat from pyrolysis to be utilized for other purposes.^48,49^ Furthermore, the use of high-temperature particulate matter filters could significantly improve the efficiency of heat exchangers and thus improve the overall energy balance of the facility.

The magnitude of the quantified climate benefits depends on the selected methodology of life cycle impact quantification. Although more uncertain than the standard descriptive approach, the prospective LCA shows that the system of sludge pyrolysis has the potential for an even better performance in the future. Over time, advancements in technology, knowledge, and social or policy goals are expected to benefit long-lived facilities, such as the sludge pyrolysis system. The analyzed prospective scenario corresponds to the middle-of-the-road expectation to how the global socioeconomic system will evolve. On the other hand, if the system were to shift toward stronger climate policies, the benefits for the balance of this pyrolysis system would be even higher, and vice versa. The decision to consider biogenic carbon affects the results and reduces the quantified benefits, yet it does not affect the overall conclusions. However, considering biogenic carbon to be compensated only upon forest regrowth shifts the results significantly, depending on the rotation period. In combination with the overall uncertainty in the results, the conclusion that the system provides climate benefits over the assessment period cannot be affirmed with high confidence from this perspective.

Prospective dynamic assessment provides a more detailed picture of the environmental impacts of the system. It might be argued that scenario predictions of the future, which are an integral part of this approach, bring more uncertainty to the results. And it is certainly true that in the future there will be many yet unknown unknowns that could largely affect the environmental balance of any system. On the other hand, those uncertainties are a part of the system regardless, only the standard LCA methodology largely ignores them. Ignoring the incertitude does not make it go away though. Hence, it is arguably better to attempt to grapple with it. Similarly, exploring the range of possibilities with various methodological approaches, including the treatment of biogenic carbon, provides further robustness to the conclusions of any study. Acknowledging that applying the full array of methodological options is more demanding than the standard methodology, it is certainly not necessary for all purposes of LCA studies. Yet it seems undeniable that, *ceteris paribus*, more detailed and robust assessments are always preferable.

## 5 Conclusions

This study applied the Life Cycle Assessment methodology to evaluate the environmental impacts of the sludge pyrolysis unit. The results show that sludge drying is the main driver of the environmental impacts in the system, while biochar soil application brings significant environmental benefits. Overall, the system was found to bring significant climate benefits over the 100 year horizon, and this conclusion is valid across the range of uncertainty in the results. Further detail and robustness to the assessment were added by employing the prospective and dynamic LCA modeling approach. With the evolution of the background socioeconomic system, the climate balance of the system is expected to improve over the years. The dynamic analysis shows that the system provides significant temporal carbon capture, which gradually decreases as biochar decomposes in the soil. Taking two perspectives on how to consider biogenic carbon emissions shows how the results can be affected by methodological decisions. Sludge pyrolysis and the application of biochar have been a widely researched topic in the past years, including their life cycle environmental impacts.^16,50^ This study pushes methodological boundaries and provides an even deeper and more detailed understanding of the dynamic evolution of climate impacts across the entire lifetime of operation of the facility.

## Supplementary Material



## Data Availability

The inventory data and full results are available in the electronic Supporting Information.

## Notes

Supplementary table containing inventory data and full results (XLSX)
